# How flexible leadership ability affects manufacturing enterprises’ digital transformation willingness: The role of innovation commitment and environmental dynamics

**DOI:** 10.1371/journal.pone.0288047

**Published:** 2023-11-02

**Authors:** Jianxin Zhu, Yu Jin

**Affiliations:** School of Economics and Management, Harbin Engineering University, Harbin, Heilongjiang Province, China; Universiti Kebangsaan Malaysia, MALAYSIA

## Abstract

Existing studies have recognized the significance of leadership ability on enterprises’ digital transformation. However, few of them pay attention to the mechanism of flexible leadership ability (FLA) on digital transformation willingness (DTW). This study aims to explore the influence mechanism of FLA on DTW based on the ability-behavior-purpose logical framework. Survey data is collected from 509 large and medium-sized manufacturing enterprises in China, and multiple regression and PROCESS Macro methods are used for hypothesis testing. This study mainly discusses the impact of FLA on DTW, as well as the mediating role of innovation commitment (IC) and the moderating role of environmental dynamics (ED). Results show that FLA promotes DTW directly (β = 0.574, p<0.001) and indirectly, that is, through IC (the main effect decreased from (β = 0.574, p<0.001) to (β = 0.40, p<0.001). Additionally, the moderating role of ED affects the direct path of FLA on DTW (β = 0.167, p<0.001) as well as the two indirect paths (β = 0.196, p<0.001; β = 0.104, p<0.01). The findings contribute to the advancement of flexible leadership theory, and provide practical advice for enterprises on how to implement digital transformation.

## 1. Introduction

Digital transformation is an effective strategy for Chinese manufacturing enterprises to achieve high-quality development [1]. More and more manufacturing enterprises start to place focus on digital transformation to achieve flexible manufacturing and satisfy consumers’ customized requests through digital technology, equipment upgradings, and other methods [2]. However, the high failure rate is causing enterprises to stagnate, drastically dampening their enthusiasm, and decreasing enterprises’ digital transformation willingness. According to Accenture’s 2021 Chinese Enterprise Digital Transformation Index report, only 9% of Chinese enterprises have achieved significant transformation results, indicating a high failure rate in enterprise transformation. The primary reason is that enterprises focus more on the selection and use of digital technology resources during the transformation process. Failing to recognize the value of leadership ability in addressing transformation management issues can lead to a range of problems [3]. For instance, mismatch of knowledge resources, inaccurate identification of transformation opportunities, and low efficiency of digital applications. Existing studies show that flexible leadership ability can promote enterprise reform by increasing enterprises’ adaptability to environmental changes [4]. Therefore, it is of great significance to investigate how flexible leadership ability can increase enterprises’ digital transformation willingness. Exploring this issue will benefit academics studying innovation and strategic management, as well as enterprises undergoing digital transformation.

Scholars have investigated the connection between managers and organizational changes from various perspectives. From the perspective of managers’ characteristics, scholars emphasize the importance of managers’ professional, educational background and experience in promoting the efficiency of enterprise transformation [5, 6]. From the perspective of managers’ cognition, scholars emphasize the positive influence of managers’ cognitive flexibility on enterprises’ capacity to recognize transformation opportunities and adapt to environmental changes [7]. From the perspective of managers’ ability, researchers believe that managers’ ability, which is a combination of decision-making, creativity, and management skills [8], is the key to enhancing enterprise transformation efficiency.

As the digital age continues to evolve, enterprises face a digital environment that grows ever more complex, dynamic, and unpredictable. To maintain momentum for digital transformation, enterprise leaders need to adapt to environmental changes [9]. Yet, traditional one-dimensional leadership capabilities cannot meet the need for leadership flexibility in a digital environment [10]. To increase flexibility and adaptability, leaders need a wider range of capabilities, including predictive [11], experiential and technical [12], and managerial [13] capabilities. While some studies have recognized the importance of leadership capabilities in enterprises’ digital transformation [10], they fail to specify what types of leaders are able to flexibly leverage their capabilities to strengthen enterprises’ digital transformation willingness.

The flexible leadership theory provides a theoretical basis for addressing the above issues. Flexible leadership is the ability to be flexible, adaptable, and innovative amid changing circumstances [14]. These comprehensive capabilities help increase the level of flexible production in enterprises and thus adapting more effectively to the evolving demands of the digital economy. Specifically, with flexible leadership, an enterprise can adapt to external changes by flexibly adjusting its strategic goals and resource mix [2]. And then by optimizing the input of innovation resources, the enterprise can have a wider, more flexibility space for innovation. Undoubtedly, only leadership turned into creative behavior can achieve the ultimate goal (ability-behavior-purpose). In other words, based on leaders’ cognitive biases and values, enterprises can modify strategies and allocate resources (innovation commitment) to affect organizations’ output, services, and business models [15]. Under the ability-behavior-purpose logical framework, this study will help explore the internal influence mechanism of flexible leadership ability on digital transformation willingness, and the influence varies as the environmental dynamic changes [16].

In conclusion, this study aims to explore the internal influence mechanism of flexible leadership ability on digital transformation willingness. Specifically, based on the ability-behavior-purpose framework, this study explores the relationship between flexible leadership ability, innovation commitment, and digital transformation willingness, as well as the boundary role of environmental dynamics. The findings contribute to the advancement of flexible leadership theory, and provide practical advice for enterprises on how to implement digital transformation.

## 2. Hypothesis development

### 2.1 Flexible leadership ability and digital transformation willingness

Based on the Process Perspective and relevant research, this study defines digital transformation willingness as the extent to which traditional manufacturing enterprises improve their business processes by integrating digital technologies to meet customers’ personalized needs [17, 18]. Digital technologies include big data, artificial intelligence, cloud computing, and digital twins. Business process includes production process, business model, and product service. Leadership capability determines the level of control over transformation resources and technologies, so it has an important impact on digital transformation willingness. Yukl first proposed the concept of flexible leadership ability in 2004 [19]. He pointed out that flexible leadership is the ability to balance competing demands, and to seek coordination and alignment between crisscrossing management layers and subsystems [14]. Flexible leadership ability can significantly increase manufacturing enterprises’ digital transformation willingness during the digital transformation process. Because flexible leadership ability can effectively address critical issues that arise as circumstances of manufacturing enterprises change, such as mismatched knowledge resources, erroneous assessment of transformation opportunities, and ineffective digital application. The details are as follows:

First, flexible leadership ability can help enterprises implement a flexible production mode, which aligns their allocation of stock resources with the continuously changing demands. Effective resource allocation supports the “soft landing” of digital transformation [19]. Second, the practical experience of flexible leadership enables effective market opportunity and risk assessment. New opportunities drive the application of digital technologies in business processes, products, and services [20] and help exploit the cost-cutting and efficiency-enhancing effects of digital technologies, thereby increasing enterprises’ digital transformation willingness. Third, thanks to systematic thinking, open-mindedness, and a learning mindset, leaders with flexible leadership ability tend to draw on experiences and keep learning up-to-date knowledge [21]. This lays the foundation for enterprises to make wise decisions on how and where to apply each digital technology. Accordingly, we propose the following:

H1: Flexible leadership ability has a positive impact on digital transformation willingness.

### 2.2 The mediating role of innovation commitment

Innovation commitment refers to enterprises’ willingness to allocate resources and conduct innovation activities, based on leaders’ cognitive biases and values [22]. This willingness can influence enterprises’ behavior to change their product, service, and business models, and therefore serves as a “soft resource” for leaders to encourage enterprise innovation [15]. Innovation commitment has two dimensions: R&D investment and innovation atmosphere. The former examines whether organizations dedicate sufficient resources to innovation activities like product development, while the latter focuses on how much leaders are involved in various innovative activities [23].

Flexible leadership ability refers to the ability to recognize opportunities and quickly realign resources in response to the changing needs of consumers [20]. Therefore, flexible leadership may actively support an enterprise’s innovation commitment. First, by flexibly adjusting R&D investment, flexible leadership ability activates the potential of quality digital resources [24], encouraging enterprises to develop innovative products under digital production models to meet customers’ personalized needs. Second, flexible leaders are more tolerant of innovation-related risks, encouraging employee innovation, thereby assisting enterprises to create a positive atmosphere for digital innovation.

H2: Flexible leadership has a positive impact on innovation commitment.

H2a: Flexible leadership ability has a positive impact on R&D investment.

H2b: Flexible leadership ability has a positive impact on innovation atmosphere.

From the perspective of resource allocation, flexible leadership can provide guarantee for digital production by improving enterprises’ use efficiency of innovative resources and creating a positive innovation atmosphere.

First, flexible leadership ability can increase digital resource efficiency through flexible resource allocation [19]. Effective use of digital resources helps enterprises overcome resource constraints and enhances enterprises’ digital transformation willingness. Second, the adaptive and innovative ability of flexible leadership help enterprises build a positive innovation atmosphere [15]. A favorable innovation environment encourages employee participation in digitization innovation, which boosts innovation efficiency and enhances enterprises’ digital transformation willingness. As a result, flexible leadership skills might indirectly increase manufacturing enterprises’ digital transformation willingness by transforming into innovation commitment (ability-behavior-purpose). Accordingly, we propose the following:

H3: Innovation commitment mediates the relationship between flexible leadership ability and digital transformation willingness.

H3a: R&D spending mediates the relationship between flexible leadership ability and digital transformation willingness.

H3b: Leaders’ support mediates the relationship between flexible leadership ability and digital transformation willingness.

### 2.3 The moderating role of environmental dynamics

Environmental dynamics, such as technological fluctuation, market fluctuation, and competitive intensity, measure the extent and predictability of enterprises’ external environment changes [25, 26]. Among them, technology fluctuation emphasizes how fast new technological changes happen or how responsively governments enact according rules. Market fluctuation describes the extent to which customer preferences shift. Competitive intensity is how fierce the competition is among enterprises in an industry providing the same or similar products and services.

Environmental dynamics are the catalyst that promotes enterprises’ innovative behavior [27]. It runs through every aspect of enterprises’ innovation activities. In a highly dynamic environment, product upgrades are frequent, customer preferences are unpredictable, and market competition is fierce. Environmental changes push flexible leadership to address these risks by increasing resource investment and assigning strategic resources wisely. And internal consensus is easier to reach under the flexible production mode, prompting flexible leadership to encourage employee creativity in response to changing demands. In a low dynamic environment, customer demands and market competition are relatively stable and predictable [28]. Leaders can help enterprises achieve steady profitability by applying existing technologies and strategies and are more difficult to play the positive role of flexible leadership ability in promoting innovation commitment. Accordingly, we propose the following:

H4: Environmental dynamics moderate the relationship between flexible leadership ability and innovation commitment positively.

H4a: Environmental dynamics moderate the relationship between flexible leadership ability and R&D expenditure positively.

H4b: Environmental dynamics moderate the relationship between flexible leadership ability and innovation atmosphere positively.

According to the Contingency Theory, under different environmental dynamics, the impact of flexible leadership ability on digital transformation willingness is different [17]. First, higher environmental dynamics provide more innovation opportunities for enterprises and therefore flexible leadership ability can exert its positive influence on digital transformation willingness more easily. Specifically, enterprises with forward-looking leaders are able to foresee the future trends of digital technology, clarify their digital development strategies, and therefore develop stronger digital transformation willingness. Additionally, flexible leadership ability can help enterprises reorient their digital development, redefine organizational processes and business models when facing new opportunities, which helps enterprises strengthen their digital transformation willingness. Second, when the environmental dynamics are low, namely corporate development is relatively stable and the scope for resource adjustment is relatively small, enterprises operate efficiently thanks to stable strategic decisions. Enterprises do not have a great need for flexible leadership ability. At the moment, pursuing flexible leadership ability excessively will increase the cost of enterprise operation and decision-making. Accordingly, we propose the following:

H5: Environmental dynamics have a positive moderating effect between flexible leadership ability and digital transformation willingness.

Manufacturing enterprises’ innovation commitment is influenced by both flexible leadership ability and environmental dynamics. Two perspectives can be used to explain this. First, from a profit-maximizing perspective, whether flexible leadership ability can promote an enterprise’s innovation commitment depends on the benefits and costs that increasing innovation commitment brings to the enterprise. Second, from a resource-matching perspective, a higher level of innovation commitment is jointly driven by market demand, customer preference, technological change, and other factors. Matching resources and technologies are allocated to better respond to customers’ individual needs. Mihardjo et al. (2017) believes that the digital model is a more effective, dynamic mode of operation that leaders seek in response to external environmental changes [10]. In other words, leadership flexible ability and environmental dynamics jointly influence the digital transformation of enterprises. Based on the previous hypotheses, we assume that environmental dynamics strengthen the impact of flexible leadership ability on innovation commitment and digital transformation willingness. Meanwhile, innovation commitment plays a mediating role between flexible leadership ability and digital transformation willingness. As a result, we infer that the interaction between environmental dynamics and flexible leadership ability not only affects innovation commitment directly, but also affects digital transformation willingness indirectly through innovation commitment. Accordingly, we propose the following:

H6: Innovation commitment mediates the moderating effect of environmental dynamics.

H6a: The interaction between environmental dynamics and flexible leadership ability influences digital transformation willingness through the mediating effect of R&D investment.

H6b: The interaction between environmental dynamics and flexible leadership ability influences digital transformation willingness through the mediating effect of innovation atmosphere.

**Fig 1:**
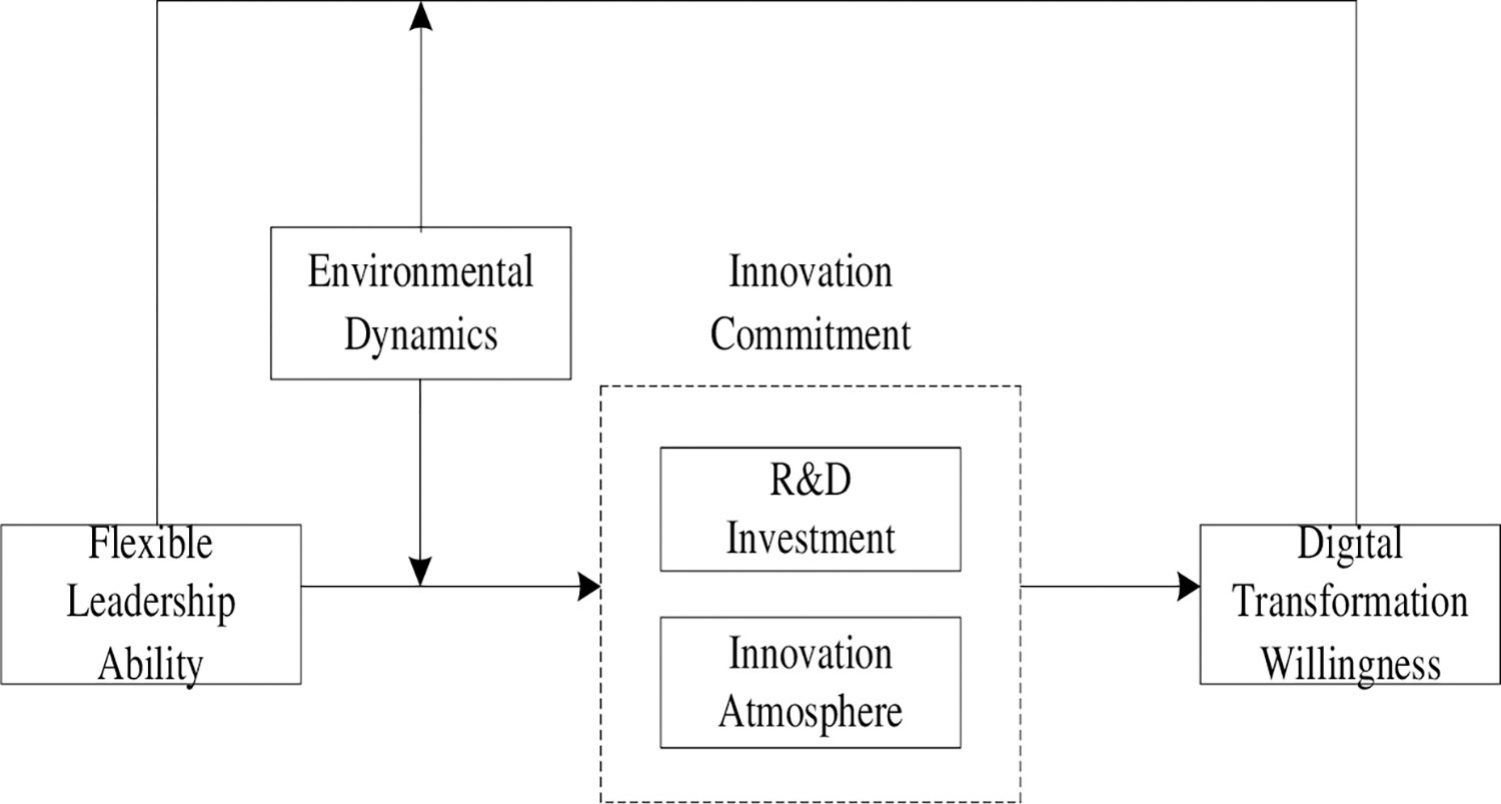
Theoretical model.

The theoretical model is shown in Fig 1.

## 3. Methodology

### 3.1 Data collection

This paper focuses on large and medium-sized manufacturing enterprises in China. Data were collected by questionnaires using random sampling. Large and medium-sized manufacturing enterprises are chosen for two reasons. First, they have comparative technological and resource advantages. Second, small and micro enterprises have limited transformation willingness due to resource deficiencies and their indistinct role in the industrial chain.

The empirical data were collected through a questionnaire survey. The questionnaires were distributed to middle and senior managers of large and medium-sized manufacturing enterprises in 30 Chinese provinces with industries covering communications equipment, special equipment, biomedicine, transportation, and others. Based on previous research, the questionnaire was modified to reflect the national circumstances of China and the objectives of this study. First, by analyzing authority scale, translating and comparing indicators, we obtained a semi-open questionnaire. Second, 45 EMBA and MBA students who are middle and senior managers of manufacturing enterprises were selected to assess the questionnaire in December 2021. According to the survey results, the technical terms and repeated items were adjusted to form a formal questionnaire. Details are given in S1 Appendix. Finally, 1200 questionnaires were distributed in two ways from January to June 2022. 600 anonymous questionnaires were faxed and e-mailed to the middle and senior managers of school-enterprise cooperative enterprises. The other 600 online questionnaires were distributed through a recurring questionnaire based on social relationships. A total of 565 questionnaires were collected with a response rate of 47.10%. After 56 questionnaires were eliminated due to incomplete or regular responses, 509 questionnaires remained valid, leaving the final effective recovery rate at 42.40%. Sample description is shown in Table 1. As suggested by Armstrong and Overton [29], a t-test was conducted to check data acquisition deviation from the two channels. The t-test showed Sig = 0.721>0.05, indicating no significant difference between the two sets of data.

**Table 1:** Sample description.

Sample characteristics	Measurement index	Effective recovery	Effective recovery rate %
Business income	>20 million≦50 million	125	24.51%
	>50 million≦400 million	258	50.59%
	>400 million≦5 billion	86	16.86%
	>5 billion≦50 billion	36	7.06%
	> 50 billion	5	0.98%
Establishment years	>5 years≦10 years	110	21.57%
	>10 years≦15 years	174	34.12%
	>15 years≦20 years	100	19.61%
	>20 years≦25 years	109	21.37%
	>25years	17	3.33%
Industry distribution	Electronic equipment manufacturing (communications equipment, computer, etc.)	165	32.35%
	Special equipment manufacturing (environmental protection, postal service, etc.)	136	26.67%
	Biomedical and medical equipment manufacturing	71	13.92%
	Transportation equipment manufacturing	58	11.37%
	Others	80	15.69%

Note: Large enterprises≥4 billion, medium-sized enterprises≥20 million (business income division standard); Business income in RMB¥.

### 3.2 Variables

Flexible leadership ability, innovation commitment, digital transformation willingness, and environmental dynamics are the four variables included in the research model. The measurement items were adjusted according to the research maturity scale, the national conditions of China, and the objectives of this study. The items were measured using a 5-point Likert scale.

Among them, based on the research of Kaiser et al. [14] and the unique conditions of Chinese manufacturing enterprises’ innovation practices, flexible leadership ability was measured using five indicators, such as increasing opportunities. Following the research of Brentani and Kleinschmidt [23], innovation commitment is divided into two dimensions: R&D investment and innovation atmosphere. Three indicators, including the percentage of R&D expenditure, are used to measure R&D investment. Three indicators, such as the leader’s innovation risk tolerance, are used to measure innovation atmosphere. According to Riemenschneider et al., digital transformation willingness is measured by four indicators, such as the degree of concern about the digitalization of key business processes [30]. Based on the research by Permana et al., four indicators, such as the degree of change in customer demands, can be used to measure environmental dynamics [31].

To minimize the impact of other variables on the dependent variable, we use corporate income, years of establishment, and industry distribution as control variables based on previous research [32]. We also use Jian and Wong’s method, which includes dummy variables in four key industries with higher digital transformation rates as control variables [33], to remove the effect of industry variation on digital transformation willingness. The value is 1 if the enterprise is in this industry, otherwise 0. It is in other industries if the four dummy variables are all marked as 0.

Three journal test methods are used to test the effect of homology deviation on the questionnaire. ① Harman’s single-factor test is used to analyze the homology bias problem [34], 30.958% is well below the critical value of 50%, which preliminarily confirms that there is no significant homologous bias problem [35]. ② AMOS21.0 is used to introduce a global factor to form a two-factor model with the original local factor. Compared to the 4-factor model, the reduced value of RMSEA and the increased value of the fit index of the 2-factor model are both below the critical value (0.05, 0.10). Table 2 shows that the homologous bias is not significant [36]. ③ Finally, according to the marker variable test of confirmatory factor analysis (CFA) by Tang and Wen [37], this paper uses environmental dynamics as a marker variable to test homology bias, and the results show no significant effect.

**Table 2:** Overall fitting results of the model.

Factor model	CMIN/DF	RMSEA	GFI	AGFI	IFI	TLI	CFI
1-factor model	10.140	0.134	0.787	0.716	0.719	0.671	0.718
2-factor model	9.686	0.131	0.792	0.719	0.736	0.687	0.735
3-factor model	6.767	0.107	0.838	0.776	0.829	0.792	0.828
4-factor model	2.516	0.055	0.949	0.928	0.956	0.945	0.956
Two-factor model	2.115	0.047	0.958	0.940	0.969	0.960	0.968
Reference range	0–5	<0.08	>0.90	>0.90	>0.90	>0.90	>0.90

1-factor model: flexible leadership ability + R&D investment + innovation atmosphere+ digital transformation willingness; 2-factor model: flexible leadership ability + R&D investment + innovation atmosphere, digital transformation willingness; 3-factor model: flexible leadership ability, R&D investment + innovation atmosphere, digital transformation willingness; 4-factor model: flexible leadership ability, R&D investment, innovation atmosphere, digital transformation willingness

## 4. Empirical results

### 4.1 Reliability, validity analysis, and correlation test

Structural equation modeling is used to test the model and hypotheses of this paper. The main reason is that structural equation modeling is suitable for exploratory and predictive research and is more effective at handling non-normal sample data [38], which is in line with the objectives of this paper.

Each variable is tested for validity and reliability using SPSS21.0. The results are shown in Table 3. Information on the measurement items for each variable is available on the Appendix page. The α coefficients of all variables vary between 0.745 and 0.934, exceeding the critical value of 0.70. The CR coefficients of all variables vary between 0.841 and 0.953, exceeding the critical value of 0.70. The above results indicate that the scale has good reliability [39]. Each indicator’s KMO value is generally greater than 0.70 and the Bartlett test is significant, indicating that it is suitable for factor analysis. The factor loadings range from 0.657 to 0.925, and the AVE values range from 0.523 to 0.836, both of which are above the critical value of 0.50. The scale therefore has good convergent validity [40].

**Table 3:** Reliability and validity analysis results.

Concept (Latent variable)	Factor loading	AVE	CR	α value	KMO Value
Flexible leadership ability	**0.749**	0.523	0.845	0.761	0.782
	**0.703**				
	**0.657**				
	**0.798**				
	**0.702**				
R&D investment	0.891	0.760	0.905	0.858	0.738
	0.893				
	0.882				
Innovation atmosphere	**0.786**	0.637	0.841	0.745	0.685
	**0.827**				
	**0.830**				
Digital transformation willingness	0.805	0.609	0.862	0.786	0.785
	0.768				
	0.763				
	0.785				
Environmental dynamics	**0.923**	0.836	0.953	0.934	0.859
	**0.899**				
	**0.925**				
	**0.910**				

The correlation coefficient of each variable is tested using SPSS21.0. The results are shown in Table 4. The correlation coefficient of each variable is between 0.042 and 0.6108, which is less than 0.70. The maximum value of VIF is less than 5, indicating that there is no serious multi-linear problem between the variables. The AVE value is greater than 0.50 and the square root is greater than the correlation coefficient between the variables, indicating that the variables have good discriminant validity [40]. In addition, we use CFA to examine the discriminant validity [41] of the indicators of each variable in this paper.

**Table 4:** Statistics and correlation coefficients.

Variable	Mean	Standard deviation	1	2	3	4	5
1. Flexible leadership ability	3.961	0.555	**0.723**				
2. R&D investment	3.580	0.929	0.314**	**0.872**			
3. Innovation atmosphere	4.061	0.673	0.513**	0.378**	**0.798**		
4. Digital transformation willingness	4.018	0.683	0.608**	0.413**	0.562**	**0.780**	
5. Environmental dynamics	3.760	1.094	0.042	0.210**	0.137**	0.046	**0.914**

Note:

***, **, and * indicate significance at 0.001, 0.01, 0.05 (bilateral) respectively, N = 509; The diagonal value is the AVE.

We examine the overall model fit to confirm the validity of the empirical test of the theoretical model. The results are shown in the 4-factor model in Table 2. It shows that the model fits well overall.

### 4.2 Regression results

The following steps are taken to test the influence mechanism and boundary conditions of flexible leadership ability on digital transformation willingness. First, the results are examined using the multi-linear regression approach. The test results of 14 regression models are shown in Tables 5 and Table 7 respectively. Second, following Hayes’ research [42], this paper uses the bootstrap method to test the significance of the mediating effect. This method can increase the validity of statistical results by compensating for the shortcomings of the stepwise regression method. The results are shown in Table 6.

**Table 5:** Regression results of the mediating effect of innovation commitment.

	Model 1	Model 2	Model 3	Model 4	Model 5	Model 6	Model 7	Model 8
	Digital transformation willingness				R&D investment		Innovation atmosphere	
Control variable								
Business income	0.170***	0.075	0.069	0.036	0.199***	0.160***	0.127**	0.048
Years of Establishment	-0.061	-0.014	-0.051	-0.023	0.027	0.047	-0.035	0.004
Communications industry	0.304***	0.151**	0.059	0.033	0.415***	0.351***	0.341***	0.214***
Special equipment	0.236***	0.133**	0.017	0.017	0.425***	0.382***	0.279***	0.194***
Biomedicine	0.259***	0.174***	0.09	0.082	0.345***	0.310***	0.216*	0.146**
Transportation	0.183**	0.125**	0.072	0.067	0.248***	0.223***	0.127*	0.079
Independent variable								
Flexible leadership ability		0.574***		0.400***		0.240***		0.475***
Mediator Variable								
R&D Investment			0.209***	0.160***				
Innovation atmosphere			0.465***	0.286***				
MAX VIF	1.257	1.068	1.203	1.321	1.259	1.068	1.259	1.068
F	7.978***	256.10******	115.203***	45.3***	16.43***	34.5***	7.38***	149.196***
R^2^	0.087	0.396	0.375	0.489	0.164	0.218	0.081	0.292
Adj.R^2^	0.076	0.387	0.365	0.480	0.154	0.207	0.070	0.282

Note:

***, **, and * indicate levels of 1%, 5%, 10% (bilateral), n = 509

**Table 6:** Mediating effect of innovation commitment on flexible leadership ability and digital transformation willingness.

Route	Effect	Correction deviation (95% CI)		Percentile (95% CI)	
		Low	High	Low	High
Flexible leadership ability → Digital transformation willingness	Total effect	0.827	1.353	0.834	1.360
	Indirect effect	0.273	1.391	0.278	1.408
	Direct effect	-0.264	0.924	-0.313	0.909

#### 4.2.1 Test of the direct effect of flexible leadership ability on digital transformation willingness

Model 1 is used to describe the influence of control variables on digital transformation willingness. Model 2 is used to explain the impact of control variables and flexible leadership ability on digital transformation willingness. As shown in Table 5, R^2^ is significantly improved. The results show that flexible leadership ability has a positive effect on digital transformation willingness, assuming H1 is accepted.

#### 4.2.2 Test of mediating effect of innovation commitment

Stepwise regression is used to test the hypotheses. First, the effect of flexible leadership ability on innovation commitment is examined. Model 5 shows the effect of control variables on R&D investment. Model 6 shows the effect of control variables and flexible leadership ability on R&D investment. As shown in Table 5, R^2^ is significantly improved, indicating that flexible leadership ability has a positive effect on R&D investment in manufacturing enterprises (β = 0.240, p<0.001), assuming H2a is accepted. Model 7 shows the effect of control variables on innovation atmosphere. Model 8 shows the effect of control variables and flexible leadership ability on innovation atmosphere. As shown in Table 5, R^2^ is significantly improved, indicating that flexible leadership ability affects the innovation atmosphere of manufacturing enterprises positively (β = 0.475, p<0.001), assuming H2b is accepted.

Second, the effect of innovation commitment on digital transformation willingness is examined. Model 3 shows the positive effect of R&D support and innovation atmosphere on digital transformation willingness (β = 0.209, p<0.001; β = 0.465, p<0.001). Finally, the role of flexible leadership ability in promoting digital transformation willingness is examined. When the mediator variable of innovation commitment is introduced into the model, the main effect decreases from (β = 0.574, P <0.001) to (β = 0.40, P <0.001) compared to Model 2. It can be tentatively inferred that innovation commitment plays a partly mediating role in the relationship between flexible leadership ability and digital transformation willingness. Further significance testing is required.

Table 6 shows the results of the 2,000 sample tests using the bootstrap method with a 95% confidence interval. The confidence interval of the total effect and the indirect effect of flexible leadership ability on digital transformation willingness does not contain 0, suggesting that there is mediation. Furthermore, a full mediating effect exists because the direct interval contains 0. Assume that H3 is accepted.

#### 4.2.3 Moderating effect of environmental dynamicsTable 7 shows the test results of the moderating effect of environmental dynamics between flexible leadership ability and innovation commitment. According to Model 9, the interaction between flexible leadership ability and environmental dynamics has a positive effect on R&D investment (β = 0.196, P<0.001). According to Model 10, the interaction between flexible leadership ability and environmental dynamics has a positive effect on the innovation atmosphere (β = 0.104, p<0.01). Assume that H4a and H4b are accepted

**Table 7:** Regression results of the moderating effect of environmental dynamics.

	Model 9	Model 10	Model 11
Variable	RDI	IA	DTW
Control variable			
Business income	0.143**	0.040	0.063
Establishment period	0.034	-0.003	-0.016
Communication industry	0.357***	0.218***	0.149**
Special equipment	0.392***	0.200***	0.141**
Biomedicine	0.293***	0.137**	0.161***
Transportation	0.215***	0.075	0.116**
Independent variable			
FLA	0.234***	0.471***	0.577***
Moderator variable			
ED	0.191***	0.117**	0.017
Interactive item			
FLA*ED	0.196***	0.104**	0.167***
MAX VIF	1.009	1.009	1.009
F	26.499***	7.728**	23.571***
R^2^	0.293	0.316	0.424
Adj.R^2^	0.280	0.304	0.413

Note:

***, **, and * denote significance at 0.001, 0.01, 0.05 (bilateral) respectively, N = 509. FLA: flexible leadership ability; ED: environmental dynamics; RDI: R&D investment; IA: innovation atmosphere; DTW: digital transformation willingness.

Table 7 shows the results of a moderating effect test of environmental dynamics between flexible leadership ability and digital transformation willingness. According to Model 11, the interaction between flexible leadership ability and environmental dynamics has a positive effect on digital transformation willingness (β = 0.167, p<0.001), assuming H5 is accepted.

In summary, based on Wen’s mediation and moderation testing process, we tentatively conclude that there is mediated moderation [43]. Additionally, conditional indirect effects and indicators (difference and index) under various levels of environmental dynamics (Mean1SD) are obtained in this study using a PROCESS Macro program and the bootstrap method [44]. These indicators can be used to determine whether environmental dynamics have a mediated moderating effect. The results are shown in Table 8.

**Table 8:** Regression results of the mediated moderating effect of environmental dynamics.

Mediation		Conditional indirect effect			Mediated moderation (Judge Index)			
variable	ED	Mediating	Standard	Confidence	DV	Confidence	Index	Confidence
		effect	error	interval		interval		interval
R&D investment	Low value	0.016	0.02	[-0.021,0.049]	0.094	[0.042,0.163]	0.043	[0.019,0.075]
	High value	0.110	0.03	[0.058,0.175]				
Innovation atmosphere	Low value	0.150	0.04	[0.073,0.213]	0.074	[0.003,0.183]	0.033	[0.002, 0.084]
	High value	0.224	0.05	[0.133,0.321]				

Note: Difference = high value-low value; 95% confidence interval; ED: environmental dynamics; DV: difference value.

**Fig 2:**
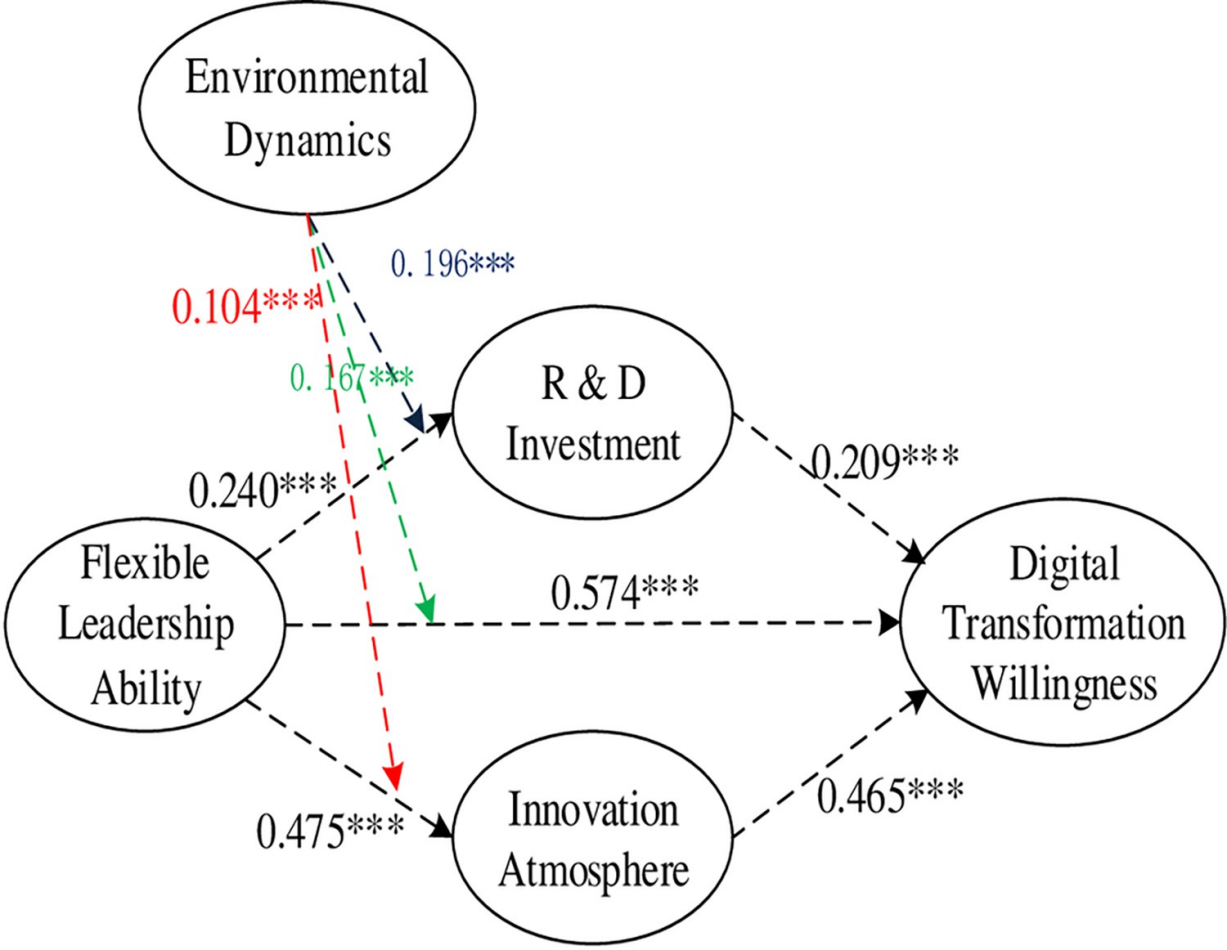
Coefficient of main path.

The path coefficients for the overall model are shown in Fig 2.

The results show that the indirect effect of R&D investment is significant (insignificant) in a high (low) dynamic environment. The indicators are significantly positive (β_Difference_ = 0.094, β_Index_ = 0.043), and the confidence interval does not include 0, assuming H6a is accepted.

**Fig 3:**
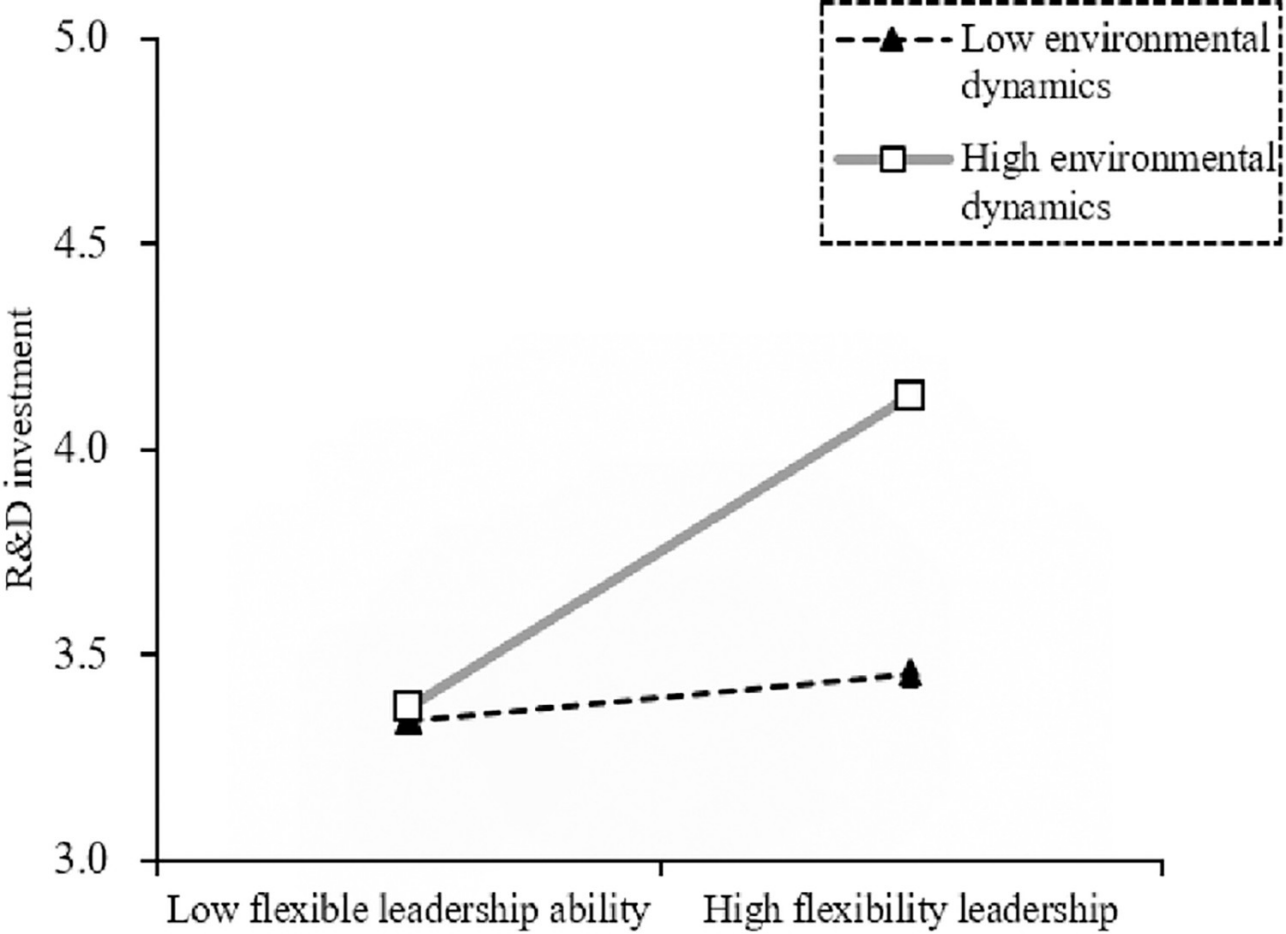
Moderating effect of environmental dynamics on the relationship between flexible leadership ability and R&D investment.

**Fig 4:**
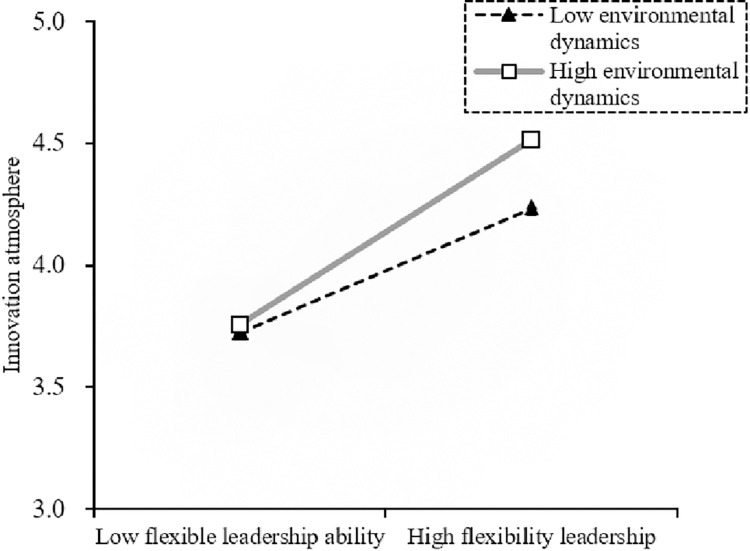
Moderating effect of environmental dynamics on the relationship between flexible leadership ability and innovation atmosphere.

**Fig 5:**
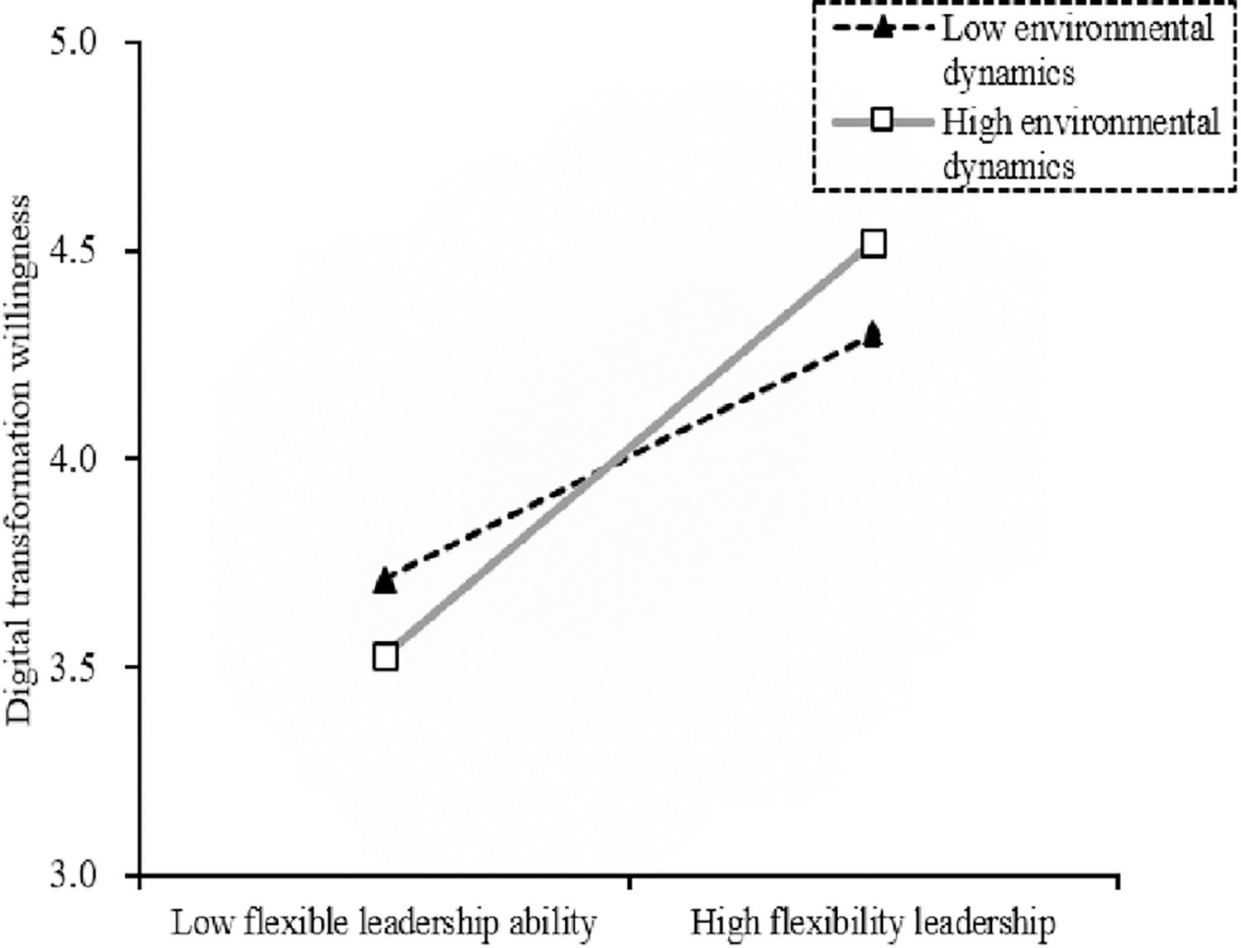
Moderating effect of environmental dynamics on the relationship between flexible leadership ability and digital transformation willingness.

The results show that the indirect effect of innovation atmosphere is always significant in a high (low) dynamic environment. But the judgment indicators are significant (β_Difference_ = 0.074, β_Index_ = 0.033) and the confidence interval does not include 0, indicating that the mediated moderating effect of environmental dynamics is supported (H6b is accepted). The detailed results are shown in Figs 3–5.

## 5. Conclusions and implications

### 5.1 Conclusions

Based on the ability-behavior-purpose logical framework, this paper primarily analyzes the influence mechanism of flexible leadership ability on manufacturing enterprises’ digital transformation willingness. Specifically, this paper examines the mediating role of innovation commitment and the moderating role of environmental dynamics in this process from the perspective of flexible leadership theory. Flexible leadership ability can directly enhance manufacturing enterprises’ digital transformation willingness, according to an empirical study using data from Chinese manufacturing enterprises. Flexible leadership ability can also indirectly enhance manufacturing enterprises’ digital transformation willingness through innovation commitment. The interaction between flexible leadership ability and environmental dynamics not only directly influences innovation commitment, but also indirectly influences digital transformation willingness through the mediating role of innovation commitment. The findings contribute to the advancement of flexible leadership theory, and provide practical advice for enterprises on how to implement digital transformation.

### 5.2 Theoretical implications

This paper focuses on the significant impact of non-technical elements, such as leadership, on enterprise reform. To achieve the ultimate goal (willingness), leadership must be turned into innovative behavior. That is to say, the ability-behavior-purpose logical framework is the foundation of this paper. This study makes the following contributions to existing literature:

First, this paper enriches the research on the previous variables of digital transformation willingness based on the flexible leadership theory. Although existing literature has realized that non-technical factors like leadership have a more significant impact than technical factors [45] when enterprises face the challenge of change. And the ability of leaders to predict and plan for digital technology evolution can help enterprises identify the value of digital transformation and improve their environmental resilience [46]. However, existing research has not clarified which type of leadership is more conducive to enterprises’ flexibility in coping with environmental uncertainty, and thus enhancing their transformation willingness. This paper complements the empirical research on the impact of flexible leadership theory on transformation willingness and supports Burke et al.’s argument that flexible leadership ability is essential for an enterprise’s digital transformation [47].

Second, we determine the crucial mediating role of innovation commitment using the ability-behavior-purpose logical framework. This is consistent with Ko et al.’s view that IT cannot effectively drive digital transformation in an enterprise without management commitment [48]. This means that the exploration of new areas requires managers to adjust resource inputs dynamically [49]. Otherwise, flexible leadership ability cannot be fully utilized to meet the needs of digital transformation. Meanwhile, it compensates for the shortcomings of the technology-driven view, which emphasizes that IT cannot directly initiate enterprise transformation to create value. Opportunities that IT offers need to be identified first by leadership based on environmental changes [50]. Then, leaders will push enterprises to align resources dynamically to support digital transformation. The result contributes to a deeper theoretical understanding of the realization path of transformation willingness.

Third, we examine the significance of environmental dynamics as a boundary condition of the ability-behavior-goal logical framework from a situational leadership perspective. This conclusion deepens the understanding of Yeows et al.’s view that flexible leadership ability will be more effective in environments with high environmental dynamics. So, enterprises can use flexible leadership ability to drive successful transformation by identifying opportunities, adjusting resources, and avoiding strategic resource misalignment [51]. In addition, this paper confirms that the interaction between flexible leadership ability and environmental dynamics directly affects innovation commitment and indirectly affect digital transformation willingness through the mediating effect of innovation commitment. According to scholar Schilke 26], these conclusions are accurate. The results extend the application of flexible leadership theory and situational leadership theory in the digital transformation of traditional manufacturing enterprises.

### 5.3 Managerial implications

The findings provide guidance for enterprises to strengthen their digital transformation willingness by improving flexible leadership ability.

First, improving leaders’ flexibility ability is essential to improving manufacturing enterprises’ digital transformation willingness. Leaders should cultivate their capacity for innovation, diversity, and decision-making. Developing these skills can increase a leader’s adaptability (“flexible intelligence”), and the improvement of flexible ability can help promote enterprises’ digital transformation willingness. And then, to expand flexibility space, leaders need to develop personal charisma and leadership ability. Extensive flexibility space can reduce the impact of uncertainty, thus contributing to enterprises’ digital transformation.

Second, enterprises should adjust their innovation commitments dynamically according to the stage of digital transformation. Specifically, the risk is relatively high in the early stage and calls for the “transform progressively while planning for the whole” strategy. Enterprises should change one step at a time while maintaining its initial competitive advantages. Gradual changes are achieved by increasing resource investment along the process or interacting dynamically with the feedback. In the mature stage, enterprises should integrate their overall development strategy with the transformation by increasing their R&D spending on new resources for business processes, production models, products, and services. This helps create a “Matthew effect” in the digital transformation of enterprises.

Third, manufacturing enterprises should be encouraged to initiate digital transformation by constantly monitoring changes in the external environment. One, by adapting to external environment changes, the potential of flexibility can be maximized. By using flexibility ability, enterprises can assign the investment in innovation commitments effectively and provide sufficient resources to support digital transformation. Two, enterprises will be more sensitive to the industrial environment. In addition to the dynamics of their immediate competitors, enterprises need to keep track of the upstream and downstream enterprises in the supply chain. Through the whole life cycle and all-around linking of software and hardware resources, enterprises can develop an effective and intelligent supply chain and establish a clearer path for digital transformation.

### 5.4 Limitations and future research

This paper enriches existing literature, but still has the following shortcomings. First, it only covers the manufacturing industry and lacks a comparative analysis of different industries, which probably will be the focus of future research. Second, this paper focuses on the influence of leaders’ ability on transformation willingness. Future research can expand the range of factors influencing digital transformation willingness to business capacities, government policies, etc. Third, to improve the generalizability of conclusions, future research could increase the sample size and compare the variations in different nations.

## Supporting information

S1 Appendix (DOCX)

S1 Data (SAV)

S1 File (DOCX)
