# Supplementary material for: How flexible leadership ability affects manufacturing enterprises’ digital transformation willingness: The role of innovation commitment and environmental dynamics
Source: PLoS One. 2023 Nov 2;18(11):e0288047. doi: 10.1371/journal.pone.0288047 (PMC10621973; doi:10.1371/journal.pone.0288047)
Supplement: S1 Appendix — (DOCX) [file pone.0288047.s001.docx]

**Appendix:**

**Measurement item**

| concept（Latent variable） | Item（Explicit variable） |
| --- | --- |
| Flexible Leadership Ability | FLA 1 Adjust decisions in a timely manner based on changing environmental information |
|  | FLA 2 Change behavior in appropriate ways as the situation changes |
|  | FLA 3 Apply experiential learning quickly and successfully to your new job |
|  | FLA 4 Take advantage of opportunities by balancing the right skill sets |
|  | FLA5 Widely accept different ideas within the organization |
| R & D Investment | R&D 1 Proportion of R & D expenditure in total enterprise revenue |
|  | R&D 2 Proportion of total population engaged in R&D activities |
|  | R&D 3 Those with master's degree or above are involved in R&D activities |
| Innovation Atmosphere | IA 1 The degree to which leaders tolerate innovation risk |
|  | IA 2 The extent to which an open innovation culture is encouraged |
|  | IA 3 The degree to which employees are motivated to innovate |
| Digital Transformation Willingness | DTW 1 Enterprises pay attention to the extent to which the key business process is digitized |
|  | DTW 2 The extent to which the enterprise promotes the adoption of key digital technologies in its products and services |
|  | DTW 3 The degree to which an enterprise uses flexible production mode for production management |
|  | DTW 4 The degree to which enterprises are willing to undertake digital transformation |
| Environmental Dynamics | ED 1 Customer market demand change degree |
|  | ED 2 Changes in product / service characteristics provided by competitors |
|  | ED 3 The degree of change in the level of technological progress |
|  | ED 4 Government technical policy’s update speed change degree |
