# Supplementary material for: How flexible leadership ability affects manufacturing enterprises’ digital transformation willingness: The role of innovation commitment and environmental dynamics
Source: PLoS One. 2023 Nov 2;18(11):e0288047. doi: 10.1371/journal.pone.0288047 (PMC10621973; doi:10.1371/journal.pone.0288047)
Supplement: S1 File — (DOCX) [file pone.0288047.s003.docx]

**Fig 1. Theoretical model**

**Fig 2. Coefficient of main path**


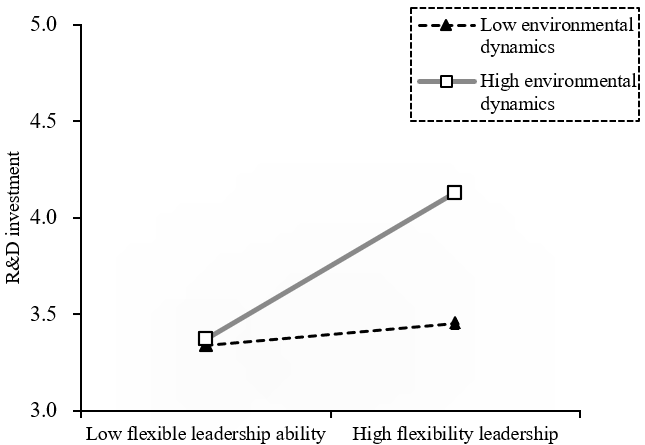


**Fig 3. Moderating effect of environmental dynamics on the relationship between flexible leadership ability and R&D investment**


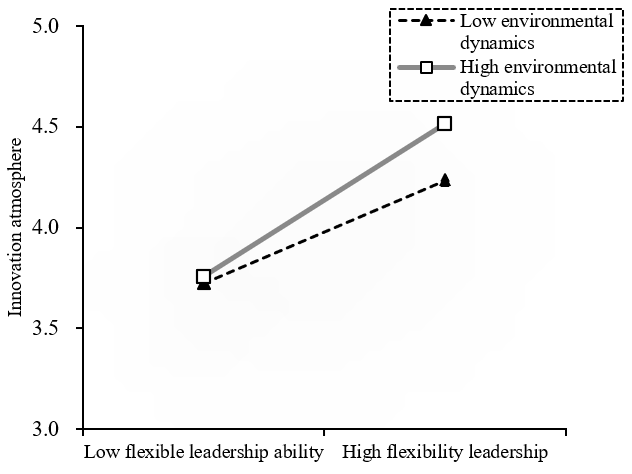


**Fig 4. Moderating effect of environmental dynamics on the relationship between flexible leadership ability and innovation atmosphere**


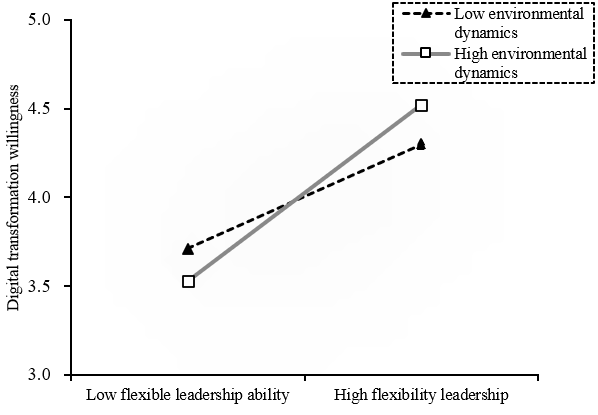


**Fig 5. Moderating effect of environmental dynamics on the relationship between flexible leadership ability and digital transformation willingness**

**Table1 Sample description**

| Sample characteristics | Measurement index | Effective recovery | | Effective recovery rate % |
| --- | --- | --- | --- | --- |
| Business income | >20 million≦50 million | 125 | | 24.51% |
|  | >50 million≦400 million | 258 | | 50.59% |
|  | >400 million≦5 billion | 86 | | 16.86% |
|  | >5 billion≦50 billion | 36 | | 7.06% |
|  | > 50 billion | 5 | | 0.98% |
| Establishment years | >5 years≦10 years | 110 | | 21.57% |
|  | >10 years≦15 years | 174 | | 34.12% |
|  | >15 years≦20 years | 100 | | 19.61% |
|  | >20 years≦25 years | 109 | | 21.37% |
|  | >25years | 17 | | 3.33% |
| Industry distribution | Electronic equipment manufacturing (communications equipment, computer, etc.) | 165 | | 32.35% |
|  | Special equipment manufacturing (environmental protection, postal service, etc.) | 136 | | 26.67% |
|  | Biomedical and medical equipment manufacturing | 71 | | 13.92% |
|  | Transportation equipment manufacturing | | 58 | 11.37% |
|  | Others | | 80 | 15.69% |

Note: Large enterprises≥4 billion, medium-sized enterprises≥20 million (business income division standard); Business income in RMB¥.

**Table 2 Overall fitting results of the model**

| Factor model | CMIN/DF | RMSEA | GFI | AGFI | IFI | TLI | CFI |
| --- | --- | --- | --- | --- | --- | --- | --- |
| 1-factor model | 10.140 | 0.134 | 0.787 | 0.716 | 0.719 | 0.671 | 0.718 |
| 2-factor model | 9.686 | 0.131 | 0.792 | 0.719 | 0.736 | 0.687 | 0.735 |
| 3-factor model | 6.767 | 0.107 | 0.838 | 0.776 | 0.829 | 0.792 | 0.828 |
| 4-factor model | 2.516 | 0.055 | 0.949 | 0.928 | 0.956 | 0.945 | 0.956 |
| Two-factor model | 2.115 | 0.047 | 0.958 | 0.940 | 0.969 | 0.960 | 0.968 |
| Reference range | 0-5 | <0.08 | >0.90 | >0.90 | >0.90 | >0.90 | >0.90 |
| 1-factor model: flexible leadership ability + R&D investment + innovation atmosphere+ digital transformation willingness; 2-factor model: flexible leadership ability + R&D investment + innovation atmosphere, digital transformation willingness; 3-factor model: flexible leadership ability, R&D investment + innovation atmosphere, digital transformation willingness; 4-factor model: flexible leadership ability, R&D investment, innovation atmosphere, digital transformation willingness | | | | | | | |

**Table 3 Reliability and validity analysis results**

| Concept (Latent variable) | Factor loading | AVE | CR | α value | KMO Value |
| --- | --- | --- | --- | --- | --- |
| Flexible leadership ability | **0.749** | 0.523 | 0.845 | 0.761 | 0.782 |
|  | **0.703** |  |  |  |  |
|  | **0.657** |  |  |  |  |
|  | **0.798** |  |  |  |  |
|  | **0.702** |  |  |  |  |
| R&D investment | 0.891 | 0.760 | 0.905 | 0.858 | 0.738 |
|  | 0.893 |  |  |  |  |
|  | 0.882 |  |  |  |  |
| Innovation atmosphere | **0.786** | 0.637 | 0.841 | 0.745 | 0.685 |
|  | **0.827** |  |  |  |  |
|  | **0.830** |  |  |  |  |
| Digital transformation willingness | 0.805 | 0.609 | 0.862 | 0.786 | 0.785 |
|  | 0.768 |  |  |  |  |
|  | 0.763 |  |  |  |  |
|  | 0.785 |  |  |  |  |
| Environmental dynamics | **0.923** | 0.836 | 0.953 | 0.934 | 0.859 |
|  | **0.899** |  |  |  |  |
|  | **0.925** |  |  |  |  |
|  | **0.910** |  |  |  |  |

**Table 4 Statistics and correlation coefficients**

| Variable | Mean | Standard deviation | 1 | 2 | 3 | 4 | 5 |
| --- | --- | --- | --- | --- | --- | --- | --- |
| 1. Flexible leadership ability | 3.961 | 0.555 | **0.723** |  |  |  |  |
| 2. R&D investment | 3.580 | 0.929 | 0.314** | **0.872** |  |  |  |
| 3. Innovation atmosphere | 4.061 | 0.673 | 0.513** | 0.378** | **0.798** |  |  |
| 4. Digital transformation willingness | 4.018 | 0.683 | 0.608** | 0.413** | 0.562** | **0.780** |  |
| 5. Environmental dynamics | 3.760 | 1.094 | 0.042 | 0.210** | 0.137** | 0.046 | **0.914** |

Note: ***, **, and * indicate significance at 0.001, 0.01, 0.05 (bilateral) respectively, N = 509; The diagonal value is the AVE.

**Table 5 Regression results of the mediating effect of innovation commitment**

|  | Model 1 | Model 2 | | Model 3 | Model 4 | Model 5 | Model 6 | Model 7 | Model 8 |  |
| --- | --- | --- | --- | --- | --- | --- | --- | --- | --- | --- |
|  | Digital transformation willingness | | | | | R&D investment | | Innovation atmosphere | |  |
| Control variable |  |  |  | |  |  |  |  |  | |
| Business income | 0.170*** | 0.075 | 0.069 | | 0.036 | 0.199*** | 0.160*** | 0.127** | 0.048 | |
| Years of Establishment | -0.061 | -0.014 | -0.051 | | -0.023 | 0.027 | 0.047 | -0.035 | 0.004 | |
| Communications industry | 0.304*** | 0.151** | 0.059 | | 0.033 | 0.415*** | 0.351*** | 0.341*** | 0.214*** | |
| Special equipment | 0.236*** | 0.133** | 0.017 | | 0.017 | 0.425*** | 0.382*** | 0.279*** | 0.194*** | |
| Biomedicine | 0.259*** | 0.174*** | 0.09 | | 0.082 | 0.345*** | 0.310*** | 0.216* | 0.146** | |
| Transportation | 0.183** | 0.125** | 0.072 | | 0.067 | 0.248*** | 0.223*** | 0.127* | 0.079 | |
| Independent variable |  |  |  | |  |  |  |  |  | |
| Flexible leadership ability |  | 0.574*** |  | | 0.400*** |  | 0.240*** |  | 0.475*** | |
| Mediator Variable |  |  |  | |  |  |  |  |  | |
| R&D Investment |  |  | 0.209*** | | 0.160*** |  |  |  |  | |
| Innovation atmosphere |  |  | 0.465*** | | 0.286*** |  |  |  |  | |
| MAX VIF | 1.257 | 1.068 | 1.203 | | 1.321 | 1.259 | 1.068 | 1.259 | 1.068 | |
| F | 7.978*** | 256.10*** | 115.203*** | | 45.3*** | 16.43*** | 34.5*** | 7.38*** | 149.196*** | |
| R^2^ | 0.087 | 0.396 | 0.375 | | 0.489 | 0.164 | 0.218 | 0.081 | 0.292 | |
| Adj.R^2^ | 0.076 | 0.387 | 0.365 | | 0.480 | 0.154 | 0.207 | 0.070 | 0.282 | |

Note: ***, **, and * indicate levels of 1%, 5%, 10% (bilateral), n = 509

**Table 6 Mediating effect of innovation commitment on flexible leadership ability and digital transformation willingness**

| Route | Effect | Correction deviation  (95% CI) | | Percentile (95% CI) | |
| --- | --- | --- | --- | --- | --- |
|  |  | Low | High | Low | High |
| Flexible leadership ability → Digital transformation willingness | Total effect | 0.827 | 1.353 | 0.834 | 1.360 |
|  | Indirect effect | 0.273 | 1.391 | 0.278 | 1.408 |
|  | Direct effect | -0.264 | 0.924 | -0.313 | 0.909 |

**Table 7 Regression results of the moderating effect of environmental dynamics**

|  | Model 9 | Model 10 | Model 11 |
| --- | --- | --- | --- |
| Variable | RDI | IA | DTW |
| Control variable |  | | |
| Business income | 0.143** | 0.040 | 0.063 |
| Establishment period | 0.034 | -0.003 | -0.016 |
| Communication industry | 0.357*** | 0.218*** | 0.149** |
| Special equipment | 0.392*** | 0.200*** | 0.141** |
| Biomedicine | 0.293*** | 0.137** | 0.161*** |
| Transportation | 0.215*** | 0.075 | 0.116** |
| Independent variable |  | | |
| FLA | 0.234*** | 0.471*** | 0.577*** |
| Moderator variable |  | | |
| ED | 0.191*** | 0.117** | 0.017 |
| Interactive item |  | | |
| FLA*ED | 0.196*** | 0.104** | 0.167*** |
| MAX VIF | 1.009 | 1.009 | 1.009 |
| F | 26.499*** | 7.728** | 23.571*** |
| R^2^ | 0.293 | 0.316 | 0.424 |
| Adj.R^2^ | 0.280 | 0.304 | 0.413 |

Note: ***, **, and * denote significance at 0.001, 0.01, 0.05 (bilateral) respectively, N=509. FLA: flexible leadership ability; ED: environmental dynamics; RDI: R&D investment; IA: innovation atmosphere; DTW: digital transformation willingness.

**Table 8 Regression results of the mediated moderating effect of environmental dynamics**

| Mediation |  | Conditional indirect effect | | | Mediated moderation (Judge Index) | | | |
| --- | --- | --- | --- | --- | --- | --- | --- | --- |
| variable | ED | Mediating | Standard | Confidence | DV | Confidence | Index | Confidence |
|  |  | effect | error | interval |  | interval |  | interval |
| R&D  investment | Low value | 0.016 | 0.02 | [-0.021,0.049] | 0.094 | [0.042,0.163] | 0.043 | [0.019,0.075] |
|  | High value | 0.110 | 0.03 | [0.058,0.175] |  |  |  |  |
| Innovation atmosphere | Low value | 0.150 | 0.04 | [0.073,0.213] | 0.074 | [0.003,0.183] | 0.033 | [0.002, 0.084] |
|  | High value | 0.224 | 0.05 | [0.133,0.321] |  |  |  |  |

Note: Difference=high value-low value; 95% confidence interval; ED: environmental dynamics; DV: difference value.
